# Recovery from Apparent Death Induced by the Inhalation of Chloroform

**Published:** 1874-10-01

**Authors:** John Rose Cormack

**Affiliations:** Paris


### RECOVERY FROM APPARENT DEATH INDUCED BY THE INHALATION OF CHLOROFORM.

By Sir John Rose Cormack, M. D., of Paris.

*From the Transactions Surg. Section Brit. Med. Assoc.*, 1874.

THE case here given was described to show the therapeutical value of inversion of the body when there is syncope from cerebral anæmia, and to aid in the elucidation of chronic chloroform poisoning.

The subject of this remarkable case was J. A., a lady's maid, an Englishwoman, aged 27. She was minutely described in the opening of the paper. The gist of the details we may sum up by saying that she was hysterical, weak and anæmic. She was admitted, under Sir John Cormack's care, to the Hartford British Hospital of Paris, on January 30, 1874, to be treated for chronic disease of the hip joint and necrosis of the femur. On May 25, Sir John resolved that she should be chloroformed, so that, painlessly to the patient, he might make a thorough exploration, and remove a loose piece of bone, which could be felt by the probe. At half-past ten on that day all was ready for the operation. The patient was placed on her left side, recumbent, and with the face quite free. The day was hot, but a slight breeze was playing. The patient lay before, and within three feet of, a large casement window. Mr. Vines administered the chloroform. This he did by placing near the patient's mouth a napkin folded as a hollow cone, and having within it a small quantity of chloroform. There was a free space of some inches between the towel and the lips of the patient. In two minutes she had passed into a calm sleep, without having spoken a word, moved a limb, or twitched a feature. Pinching the skin of the fore-arm caused only a very slight movement. The towel was now so far removed from the mouth that the inhaled chloroform vapor must have been so diluted with atmospheric air as to augment very slightly the anæsthesia. For two minutes more Sir John kept his hand on the pulse. She was then profoundly chloroformed. The breathing was natural; the pulse, though slow, was quite regular, and of fair strength. At the end of four minutes from the commencement of the inhalation of chloroform, his colleague, Dr. Herbert, joined Sir John. Just as he entered Sir John made a free incision, saying, almost simultaneously with Dr. Herbert, that the patient was too much under the influence of the chloroform. Glancing at her and feeling her pulse, Dr. Herbert replied—"Yes, but the pulse and breathing are all right." No more chloroform was given; and whilst Sir John proceeded with the operation to its completion, the window was thrown open; and Dr. Herbert tried to bring back the patient to consciousness by flapping her face and chest with a wet towel. About twenty minutes from the time at which the inhalation was commenced, Dr. Herbert announced an alarming irregularity and sinking of the pulse. At this crisis, and throughout the whole duration of the unconsciousness—except once, for a few minutes—the lips were red and the face had neither a ghastly nor a pale aspect. The pulse became more and more irregular and feeble; death seemed imminent. The body of the patient was inverted; the pulse immediately improved. The patient was maintained in an inverted position for four or five minutes; the pulse had then so much improved that danger seemed to be past. Accordingly the woman was replaced in the horizontal position, when a variety of auxiliary restorative measures were pursued. The patient was at a later stage twice inverted for a few minutes with remarkable benefit to the pulse. Many means were used when the patient was horizontal. There was partial consciousness. All seemed to be going on well, when the patient relapsed into unconsciousness, the pulse fluttered and fell, and the breathing became very weak, very slow, and occasionally jerking. At the same time, and for the first time, the lips and the cheeks became deadly pale, and for a minute or two life seemed extinct, so long an interval occurred between the respirations. Artificial respiration was employed. Under this treatment the pulse, which had become imperceptible, returned, to a certain extent, failing, however, when the artificial respiration was relinquished for a minute. At this crisis the danger seemed even more urgent than it had yet been, the respiratory function being much more profoundly compromised. Inversion was again resorted to, and again with the happiest results. A little later, vomiting came on, which seemed to bring back the patient to a certain degree of consciousness. This was the state of matters at half-past one o'clock. Between that time and half-past three she was in an improving but still very dangerous state. The relapses were frequent, but they were, with one exception, not alarming. At four o'clock she took some beef-tea. She swallowed easily. She was then in a state of semi-consciousness, in which condition she still remained on the following day. Its intensity gradually diminished; but three days and three nights had elapsed before it had entirely passed away. At the end of that time the patient had resumed her former ways in respect of food, sleep and intelligence. It is essential to state, that besides artificial respiration and inversion, many auxiliary means of resuscitation were employed, all of which must have contributed to the happy issue. In this abstract our object has been to present as fairly as possible the beneficial results of the inversion practice. The *chronic* and *cataleptiform* characters of this case of poisoning with chloroform vapor were, in the author's opinion, its most remarkable features. Several cases of a somewhat similar character were mentioned, showing that chloroform inhalation may prove fatal after an interval of many days. The author was convinced that inversion had proved useful in his case as well as in others previously described. He held, however, that all the various restorative measures which had been employed in his case contributed to recovery. He said that numerous cases are recorded in which, by means of keeping the patients warm and in the horizontal position, they have been saved by artificial respiration. The author did not recommend that, in cases of cerebral anæmia from chloroform poisoning, the inverted was preferable to the horizontal position; he only recommended the former to be used—as in the case he had described—for short intervals, and in conjunction with the persevering use of artificial breathing and the other common measures of recovery and precaution.

---

PROTECTION FROM YELLOW FEVER.—In a report on yellow fever, recently published in the United States, it is shown that this disease has never appeared in any climate at the height of 2,500 feet. In the island of Dominica, a hill-top not more than 1,500 feet high is always healthy, even when the fever is epidemic at its base. In San Domingo similar observations have been made. The highest elevation at which yellow fever has occurred in the United States is 460 feet, in Arkansas; and the medical men of this country now hold that the stratum of air infected by the poison is heavier than pure air, and therefore sinks, and they recommend that in unhealthy districts houses and hospitals should be built on tall piles, so as to be above the fever stratum. But where hills are near, the best remedy will be to carry the patients up to a height of 500 feet.

